# Diaqua­bis(*N*,*N*-diethyl­nicotinamide-κ*N* ^1^)bis­(4-methyl­benzoato-κ*O*)cobalt(II)

**DOI:** 10.1107/S1600536810013954

**Published:** 2010-04-21

**Authors:** Hacali Necefoğlu, Efdal Çimen, Barış Tercan, Emel Ermiş, Tuncer Hökelek

**Affiliations:** a Department of Chemistry, Kafkas University, 36100 Kars, Turkey; b Department of Physics, Karabük University, 78050 Karabük, Turkey; c Department of Chemistry, Faculty of Science, Anadolu University, 26470 Yenibağlar, Eskişehir, Turkey; d Department of Physics, Hacettepe University, 06800 Beytepe, Ankara, Turkey

## Abstract

In the centrosymmetric mononuclear title complex, [Co(C_8_H_7_O_2_)_2_(C_10_H_14_N_2_O)_2_(H_2_O)_2_], the Co^II^ ion is located on an inversion center. The asymmetric unit contains one 4-methyl­benzoate (PMB) anion, one *N*,*N*-diethyl­nicotinamide (DENA) ligand and one coordinated water mol­ecule. The four O atoms in the equatorial plane around the Co^II^ ion form a slightly distorted square-planar arrangement, while the slightly distorted octa­hedral coordination is completed by the two pyridine N atoms of the DENA ligands in the axial positions. The dihedral angle between the carboxyl­ate group and the attached benzene ring is 3.73 (14)°, while the pyridine and benzene rings are oriented at a dihedral angle of 77.28 (6)°. In the crystal structure, inter­molecular O—H⋯O and C—H⋯O hydrogen bonds link the mol­ecules into a two-dimensional network parallel to (001). The structure is further stabilized by π–π contacts between the pyridine rings [centroid–centroid distance = 3.544 (1) Å] and weak C—H⋯π inter­actions involving the benzene ring.

## Related literature

For niacin, see: Krishnamachari (1974 ▶), and for the nicotinic acid derivative *N*,*N*-diethyl­nicotinamide, see: Bigoli *et al.* (1972 ▶). For related structures, see: Hökelek *et al.* (1996 ▶, 2009*a* ▶,*b* ▶,*c* ▶); Hökelek & Necefoğlu (1998 ▶); Necefoğlu *et al.* (2010 ▶).
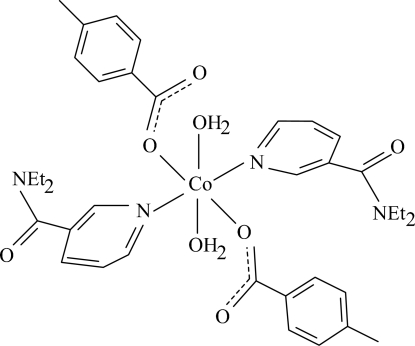


## Experimental

### 

#### Crystal data

[Co(C_8_H_7_O_2_)_2_(C_10_H_14_N_2_O)_2_(H_2_O)_2_]
*M* *_r_* = 721.70
Triclinic, 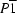

*a* = 7.2791 (2) Å
*b* = 8.5453 (2) Å
*c* = 16.0438 (4) Å
α = 84.090 (3)°
β = 77.583 (3)°
γ = 67.271 (2)°
*V* = 898.71 (4) Å^3^
*Z* = 1
Mo *K*α radiation
μ = 0.53 mm^−1^
*T* = 100 K
0.35 × 0.25 × 0.15 mm

#### Data collection

Bruker Kappa APEXII CCD area-detector diffractometer
Absorption correction: multi-scan (*SADABS*; Bruker, 2005 ▶) *T*_min_ = 0.852, *T*_max_ = 0.922
15243 measured reflections
4484 independent reflections
3821 reflections with *I* > 2σ(*I*)
*R*_int_ = 0.025

#### Refinement

*R*[*F* ^2^ > 2σ(*F* ^2^)] = 0.038
*wR*(*F* ^2^) = 0.090
*S* = 1.04
4484 reflections
234 parameters
3 restraints
H atoms treated by a mixture of independent and constrained refinement
Δρ_max_ = 0.86 e Å^−3^
Δρ_min_ = −0.55 e Å^−3^

### 

Data collection: *APEX2* (Bruker, 2007 ▶); cell refinement: *SAINT* (Bruker, 2007 ▶); data reduction: *SAINT*; program(s) used to solve structure: *SHELXS97* (Sheldrick, 2008 ▶); program(s) used to refine structure: *SHELXL97* (Sheldrick, 2008 ▶); molecular graphics: Mercury (Macrae *et al.*, 2006 ▶); software used to prepare material for publication: *WinGX* (Farrugia, 1999 ▶) and *PLATON* (Spek, 2009 ▶).

## Supplementary Material

Crystal structure: contains datablocks I, global. DOI: 10.1107/S1600536810013954/ci5076sup1.cif

Structure factors: contains datablocks I. DOI: 10.1107/S1600536810013954/ci5076Isup2.hkl

Additional supplementary materials:  crystallographic information; 3D view; checkCIF report

## Figures and Tables

**Table 1:** Selected bond lengths (Å)

Co1—O2	2.0885 (12)
Co1—O4	2.1209 (12)
Co1—N1	2.1439 (14)

**Table 2:** Hydrogen-bond geometry (Å, °) *Cg*1 is the centroid of the C2–C7 ring.

*D*—H⋯*A*	*D*—H	H⋯*A*	*D*⋯*A*	*D*—H⋯*A*
O4—H41⋯O1^i^	1.00 (2)	1.69 (2)	2.6443 (18)	160 (3)
O4—H42⋯O3^ii^	0.89 (2)	1.88 (2)	2.7557 (18)	170 (2)
C6—H6⋯O1	0.93	2.40	3.249 (3)	152
C11—H11⋯O1	0.93	2.42	3.339 (2)	168
C17—H17*A*⋯*Cg*1^iii^	0.97	2.95	3.594 (2)	125

Symmetry codes: (i) 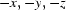; (ii) 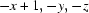; (iii) 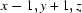.

**Fig. 1.**
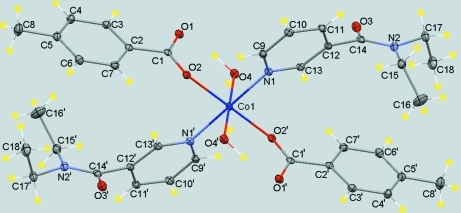
The molecular structure of the title compound, with the atom-numbering scheme. Displacement ellipsoids are drawn at the 30% probability level. Primed atoms are generated by the symmetry operator:(') -x, -y, -z.

## supplementary crystallographic information

### Comment

As a part of our ongoing investigation on transition metal complexes of nicotinamide (NA), one form of niacin (Krishnamachari, 1974), and/or the nicotinic acid derivative *N*,*N*-diethylnicotinamide (DENA), an important respiratory stimulant (Bigoli *et al.*, 1972), the title compound was synthesized and its crystal structure is reported herein.

The title complex, (I), is a crystallographically centrosymmetric mononuclear complex, consisting of two *N*,*N*-diethylnicotinamide (DENA) and two 4-methylbenzoate (PMB) ligands and two coordinated water molecules; the Co^II^ ion lies on the centre of inversion (Fig. 1). The crystal structures of similar complexes of Cu^II^, Co^II^, Ni^II^, Mn^II^ and Zn^II^ ions, [Cu(C_7_H_5_O_2_)_2_(C_10_H_14_N_2_O)_2_], (II) (Hökelek *et al.*, 1996), [Co(C_6_H_6_N_2_O)_2_(C_7_H_4_NO_4_)_2_(H_2_O)_2_], (III) (Hökelek & Necefoğlu, 1998), [Ni(C_7_H_4_ClO_2_)_2_(C_6_H_6_N_2_O)_2_(H_2_O)_2_], (IV) (Hökelek *et al.*, 2009a), [Ni(C_8_H_7_O_2_)_2_(C_6_H_6_N_2_O)_2_(H_2_O)_2_], (V) (Necefoğlu *et al.*, 2010), [Mn(C_7_H_4_ClO_2_)_2_(C_10_H_14_N_2_O)_2_(H_2_O)_2_], (VI) (Hökelek *et al.*, 2009b) and [Zn(C_7_H_4_BrO_2_)_2_(C_6_H_6_N_2_O)_2_(H_2_O)_2_], (VII) (Hökelek *et al.*, 2009c) have also been reported. In (II), the two benzoate ions are coordinated to the Cu^II^ atom as bidentate ligands, while in the other structures all ligands are monodentate.

All ligands are monodentate in (I). The four O atoms (O2, O4, and the symmetry-related atoms O2', O4') in the equatorial plane around the Co^II^ ion form a slightly distorted square-planar arrangement, while the slightly distorted octahedral coordination is completed by the pyridine N atoms of two DENA ligands (N1, N1') in the axial positions (Fig. 1). The near equality of the C1—O1 [1.257 (2) Å] and C1—O2 [1.266 (2) Å] bonds in the carboxylate group indicates a delocalized bonding arrangement, rather than localized single and double bonds. The average Co—O bond length is 2.1047 (12) Å (Table 1), and the Co1 atom is displaced out of the least-squares plane of the carboxylate group (O1/C1/O2) by 0.8823 (1) Å. The dihedral angle between the planar carboxylate group and the benzene ring A (C2—C7) is 3.73 (14)°, while that between rings A and B (N1/C9—C13) is 77.28 (6)°.

In the crystal structure, intermolecular O—H···O and C—H···O hydrogen bonds (Table 2) link the molecules into a two-dimensional network parallel to the (001). The π–π contact between the pyridine rings (N1/C9—C13) at (x, y, z) and (1-x, -1-y, -z) [centroid-centroid distance = 3.544 (1) Å] further stabilize the structure. A weak C—H···π interaction involving the benzene ring is also observed (Table 2).

### Experimental

The title compound was prepared by the reaction of CoSO_4_.7H_2_O (1.41 g, 5 mmol) in H_2_O (40 ml) and DENA (1.78 g, 10 mmol) in H_2_O (10 ml) with sodium 4-methylbenzoate (1.58 g, 10 mmol) in H_2_O (300 ml). The mixture was filtered and set aside to crystallize at ambient temperature for one week, giving pink single crystals.

### Refinement

Water H atoms (H41 and H42) were located in a difference Fourier map and refined with O–H and H···H distance restraints of 0.95 (2) Å and 1.46 (4) Å, respectively. The remaining H atoms were positioned geometrically with C-H = 0.93, 0.97 and 0.96 Å for aromatic, methylene and methyl H atoms, respectively, and constrained to ride on their parent atoms, with U_iso_(H) = xU_eq_(C), where x = 1.5 for methyl H and x = 1.2 for all other H atoms.

### Crystal data

**Table:**

[Co(C_8_H_7_O_2_)_2_(C_10_H_14_N_2_O)_2_(H_2_O)_2_]	*Z* = 1
*M**_r_* = 721.70	*F*(000) = 381
Triclinic, *P*1	*D*_x_ = 1.334 Mg m^−^^3^
Hall symbol: -P 1	Mo *K*α radiation, λ = 0.71073 Å
*a* = 7.2791 (2) Å	Cell parameters from 7347 reflections
*b* = 8.5453 (2) Å	θ = 2.6–28.3°
*c* = 16.0438 (4) Å	µ = 0.53 mm^−^^1^
α = 84.090 (3)°	*T* = 100 K
β = 77.583 (3)°	Block, pink
γ = 67.271 (2)°	0.35 × 0.25 × 0.15 mm
*V* = 898.71 (4) Å^3^	

### Data collection

**Table:**

Bruker Kappa APEXII CCD area-detector diffractometer	4484 independent reflections
Radiation source: fine-focus sealed tube	3821 reflections with *I* > 2σ(*I*)
graphite	*R*_int_ = 0.025
φ and ω scans	θ_max_ = 28.5°, θ_min_ = 1.3°
Absorption correction: multi-scan (*SADABS*; Bruker, 2005)	*h* = −9→9
*T*_min_ = 0.852, *T*_max_ = 0.922	*k* = −11→11
15243 measured reflections	*l* = −21→21

### Refinement

**Table:**

Refinement on *F*^2^	Primary atom site location: structure-invariant direct methods
Least-squares matrix: full	Secondary atom site location: difference Fourier map
*R*[*F*^2^ > 2σ(*F*^2^)] = 0.038	Hydrogen site location: inferred from neighbouring sites
*wR*(*F*^2^) = 0.090	H atoms treated by a mixture of independent and constrained refinement
*S* = 1.04	*w* = 1/[σ^2^(*F*_o_^2^) + (0.0314*P*)^2^ + 0.8168*P*] where *P* = (*F*_o_^2^ + 2*F*_c_^2^)/3
4484 reflections	(Δ/σ)_max_ = 0.001
234 parameters	Δρ_max_ = 0.86 e Å^−^^3^
3 restraints	Δρ_min_ = −0.55 e Å^−^^3^

### Special details

**Table:**

Geometry. All esds (except the esd in the dihedral angle between two l.s. planes) are estimated using the full covariance matrix. The cell esds are taken into account individually in the estimation of esds in distances, angles and torsion angles; correlations between esds in cell parameters are only used when they are defined by crystal symmetry. An approximate (isotropic) treatment of cell esds is used for estimating esds involving l.s. planes.
Refinement. Refinement of *F*^2^ against ALL reflections. The weighted *R*-factor wR and goodness of fit *S* are based on *F*^2^, conventional *R*-factors *R* are based on *F*, with *F* set to zero for negative *F*^2^. The threshold expression of *F*^2^ > σ(*F*^2^) is used only for calculating *R*-factors(gt) etc. and is not relevant to the choice of reflections for refinement. *R*-factors based on *F*^2^ are statistically about twice as large as those based on *F*, and *R*- factors based on ALL data will be even larger.

### Fractional atomic coordinates and isotropic or equivalent isotropic displacement parameters (Å^2^)

**Table:**

	*x*	*y*	*z*	*U*_iso_*/*U*_eq_	
Co1	0.0000	0.0000	0.0000	0.01216 (9)	
O1	−0.07573 (18)	0.12790 (16)	−0.20025 (8)	0.0182 (3)	
O2	0.11412 (18)	0.12158 (15)	−0.10611 (7)	0.0152 (2)	
O3	0.84302 (19)	−0.32653 (16)	−0.12244 (8)	0.0190 (3)	
O4	0.22201 (18)	0.01547 (16)	0.06192 (8)	0.0164 (3)	
H41	0.191 (4)	−0.036 (3)	0.1190 (12)	0.058 (9)*	
H42	0.211 (4)	0.117 (2)	0.0762 (16)	0.046 (8)*	
N1	0.2241 (2)	−0.23699 (18)	−0.04865 (9)	0.0138 (3)	
N2	0.8480 (2)	−0.42543 (18)	−0.24793 (9)	0.0170 (3)	
C1	0.0833 (3)	0.1274 (2)	−0.18126 (11)	0.0142 (3)	
C2	0.2508 (3)	0.1341 (2)	−0.25355 (11)	0.0148 (3)	
C3	0.2310 (3)	0.1313 (2)	−0.33787 (11)	0.0168 (3)	
H3	0.1161	0.1212	−0.3492	0.020*	
C4	0.3813 (3)	0.1434 (2)	−0.40496 (11)	0.0189 (4)	
H4	0.3655	0.1425	−0.4609	0.023*	
C5	0.5557 (3)	0.1569 (2)	−0.38953 (11)	0.0191 (4)	
C6	0.5781 (3)	0.1543 (2)	−0.30536 (12)	0.0192 (4)	
H6	0.6952	0.1603	−0.2940	0.023*	
C7	0.4276 (3)	0.1429 (2)	−0.23807 (11)	0.0165 (3)	
H7	0.4450	0.1411	−0.1822	0.020*	
C8	0.7177 (3)	0.1732 (3)	−0.46257 (13)	0.0298 (5)	
H8A	0.7231	0.1117	−0.5104	0.045*	
H8B	0.8468	0.1275	−0.4451	0.045*	
H8C	0.6861	0.2907	−0.4786	0.045*	
C9	0.1932 (3)	−0.3834 (2)	−0.03785 (11)	0.0146 (3)	
H9	0.0700	−0.3831	−0.0063	0.018*	
C10	0.3371 (3)	−0.5349 (2)	−0.07173 (11)	0.0162 (3)	
H10	0.3102	−0.6339	−0.0631	0.019*	
C11	0.5216 (3)	−0.5369 (2)	−0.11857 (11)	0.0157 (3)	
H11	0.6200	−0.6366	−0.1426	0.019*	
C12	0.5562 (3)	−0.3860 (2)	−0.12890 (10)	0.0138 (3)	
C13	0.4048 (3)	−0.2405 (2)	−0.09221 (11)	0.0146 (3)	
H13	0.4297	−0.1407	−0.0981	0.018*	
C14	0.7599 (3)	−0.3773 (2)	−0.16750 (11)	0.0147 (3)	
C15	0.7528 (3)	−0.4745 (2)	−0.30709 (11)	0.0203 (4)	
H15A	0.6229	−0.4762	−0.2769	0.024*	
H15B	0.8377	−0.5886	−0.3261	0.024*	
C16	0.7198 (4)	−0.3567 (3)	−0.38450 (15)	0.0397 (6)	
H16A	0.6555	−0.3943	−0.4203	0.060*	
H16B	0.8482	−0.3575	−0.4160	0.060*	
H16C	0.6348	−0.2435	−0.3663	0.060*	
C17	1.0549 (3)	−0.4277 (2)	−0.27880 (12)	0.0211 (4)	
H17A	1.1183	−0.5004	−0.3278	0.025*	
H17B	1.1337	−0.4769	−0.2344	0.025*	
C18	1.0623 (3)	−0.2529 (3)	−0.30397 (14)	0.0289 (5)	
H18A	1.2009	−0.2634	−0.3226	0.043*	
H18B	1.0014	−0.1803	−0.2556	0.043*	
H18C	0.9891	−0.2050	−0.3495	0.043*	

### Atomic displacement parameters (Å^2^)

**Table:**

	*U*^11^	*U*^22^	*U*^33^	*U*^12^	*U*^13^	*U*^23^
Co1	0.01068 (16)	0.01085 (16)	0.01383 (17)	−0.00297 (12)	−0.00131 (12)	−0.00186 (12)
O1	0.0155 (6)	0.0201 (6)	0.0191 (6)	−0.0067 (5)	−0.0040 (5)	0.0007 (5)
O2	0.0162 (6)	0.0136 (6)	0.0154 (6)	−0.0054 (5)	−0.0021 (5)	−0.0013 (4)
O3	0.0168 (6)	0.0199 (6)	0.0224 (7)	−0.0086 (5)	−0.0028 (5)	−0.0044 (5)
O4	0.0148 (6)	0.0155 (6)	0.0200 (6)	−0.0065 (5)	−0.0032 (5)	−0.0023 (5)
N1	0.0125 (7)	0.0129 (7)	0.0156 (7)	−0.0041 (6)	−0.0029 (5)	−0.0011 (5)
N2	0.0163 (7)	0.0158 (7)	0.0183 (7)	−0.0067 (6)	0.0004 (6)	−0.0028 (6)
C1	0.0150 (8)	0.0073 (7)	0.0173 (8)	−0.0013 (6)	−0.0024 (7)	−0.0008 (6)
C2	0.0158 (8)	0.0100 (8)	0.0166 (8)	−0.0030 (6)	−0.0023 (7)	−0.0013 (6)
C3	0.0160 (8)	0.0154 (8)	0.0181 (8)	−0.0046 (7)	−0.0036 (7)	−0.0013 (6)
C4	0.0208 (9)	0.0194 (9)	0.0149 (8)	−0.0056 (7)	−0.0030 (7)	−0.0016 (7)
C5	0.0181 (9)	0.0192 (9)	0.0182 (9)	−0.0071 (7)	0.0008 (7)	−0.0014 (7)
C6	0.0170 (8)	0.0195 (9)	0.0219 (9)	−0.0076 (7)	−0.0029 (7)	−0.0023 (7)
C7	0.0171 (8)	0.0150 (8)	0.0164 (8)	−0.0045 (7)	−0.0041 (7)	−0.0013 (6)
C8	0.0252 (10)	0.0423 (13)	0.0219 (10)	−0.0159 (10)	0.0025 (8)	−0.0027 (9)
C9	0.0125 (8)	0.0154 (8)	0.0161 (8)	−0.0056 (7)	−0.0025 (6)	−0.0001 (6)
C10	0.0172 (8)	0.0125 (8)	0.0203 (9)	−0.0065 (7)	−0.0041 (7)	−0.0010 (6)
C11	0.0153 (8)	0.0125 (8)	0.0172 (8)	−0.0028 (7)	−0.0025 (7)	−0.0024 (6)
C12	0.0134 (8)	0.0145 (8)	0.0137 (8)	−0.0054 (7)	−0.0028 (6)	−0.0001 (6)
C13	0.0147 (8)	0.0128 (8)	0.0171 (8)	−0.0058 (7)	−0.0032 (7)	−0.0002 (6)
C14	0.0139 (8)	0.0095 (7)	0.0194 (8)	−0.0031 (6)	−0.0027 (7)	−0.0001 (6)
C15	0.0228 (9)	0.0198 (9)	0.0177 (9)	−0.0075 (8)	−0.0019 (7)	−0.0034 (7)
C16	0.0536 (15)	0.0448 (14)	0.0296 (12)	−0.0251 (12)	−0.0195 (11)	0.0120 (10)
C17	0.0173 (9)	0.0182 (9)	0.0242 (9)	−0.0063 (7)	0.0048 (7)	−0.0047 (7)
C18	0.0288 (11)	0.0219 (10)	0.0342 (11)	−0.0136 (9)	0.0065 (9)	−0.0028 (8)

### Geometric parameters (Å, °)

**Table:**

Co1—O2	2.0885 (12)	C7—C6	1.388 (2)
Co1—O2^i^	2.0885 (12)	C7—H7	0.93
Co1—O4	2.1209 (12)	C8—H8A	0.96
Co1—O4^i^	2.1209 (12)	C8—H8B	0.96
Co1—N1	2.1439 (14)	C8—H8C	0.96
Co1—N1^i^	2.1439 (14)	C9—C10	1.386 (2)
O1—C1	1.257 (2)	C9—H9	0.93
O2—C1	1.266 (2)	C10—H10	0.93
O3—C14	1.238 (2)	C11—C10	1.385 (2)
O4—H41	0.996 (15)	C11—H11	0.93
O4—H42	0.889 (16)	C12—C11	1.394 (2)
N1—C9	1.342 (2)	C12—C13	1.385 (2)
N1—C13	1.341 (2)	C12—C14	1.506 (2)
N2—C14	1.340 (2)	C13—H13	0.93
N2—C15	1.465 (2)	C15—C16	1.516 (3)
N2—C17	1.474 (2)	C15—H15A	0.97
C1—C2	1.505 (2)	C15—H15B	0.97
C2—C3	1.394 (2)	C16—H16A	0.96
C3—H3	0.93	C16—H16B	0.96
C4—C3	1.387 (2)	C16—H16C	0.96
C4—C5	1.393 (3)	C17—C18	1.525 (3)
C4—H4	0.93	C17—H17A	0.97
C5—C8	1.511 (3)	C17—H17B	0.97
C6—C5	1.392 (3)	C18—H18A	0.96
C6—H6	0.93	C18—H18B	0.96
C7—C2	1.393 (2)	C18—H18C	0.96
			
O2^i^—Co1—O2	180.00 (5)	C5—C8—H8B	109.5
O2—Co1—O4	88.07 (5)	C5—C8—H8C	109.5
O2^i^—Co1—O4	91.93 (5)	H8A—C8—H8B	109.5
O2—Co1—O4^i^	91.93 (5)	H8A—C8—H8C	109.5
O2^i^—Co1—O4^i^	88.07 (5)	H8B—C8—H8C	109.5
O2—Co1—N1	88.47 (5)	N1—C9—C10	122.83 (15)
O2^i^—Co1—N1	91.53 (5)	N1—C9—H9	118.6
O2—Co1—N1^i^	91.53 (5)	C10—C9—H9	118.6
O2^i^—Co1—N1^i^	88.47 (5)	C9—C10—H10	120.5
O4—Co1—O4^i^	180.00 (8)	C11—C10—C9	119.08 (16)
O4—Co1—N1	86.58 (5)	C11—C10—H10	120.5
O4^i^—Co1—N1	93.42 (5)	C10—C11—C12	118.47 (15)
O4—Co1—N1^i^	93.42 (5)	C10—C11—H11	120.8
O4^i^—Co1—N1^i^	86.58 (5)	C12—C11—H11	120.8
N1^i^—Co1—N1	180.00 (8)	C11—C12—C14	123.16 (15)
C1—O2—Co1	126.53 (11)	C13—C12—C11	118.72 (15)
Co1—O4—H41	101.8 (17)	C13—C12—C14	117.57 (15)
Co1—O4—H42	118.7 (18)	N1—C13—C12	123.04 (16)
H41—O4—H42	101 (2)	N1—C13—H13	118.5
C9—N1—Co1	123.36 (11)	C12—C13—H13	118.5
C13—N1—Co1	118.83 (11)	O3—C14—N2	121.55 (16)
C13—N1—C9	117.81 (14)	O3—C14—C12	118.01 (15)
C14—N2—C15	124.66 (15)	N2—C14—C12	120.44 (15)
C14—N2—C17	117.31 (15)	N2—C15—C16	113.39 (16)
C15—N2—C17	118.03 (14)	N2—C15—H15A	108.9
O1—C1—O2	125.19 (16)	N2—C15—H15B	108.9
O1—C1—C2	117.52 (15)	C16—C15—H15A	108.9
O2—C1—C2	117.30 (15)	C16—C15—H15B	108.9
C3—C2—C1	120.15 (16)	H15A—C15—H15B	107.7
C7—C2—C1	121.17 (15)	C15—C16—H16A	109.5
C7—C2—C3	118.67 (16)	C15—C16—H16B	109.5
C2—C3—H3	119.7	C15—C16—H16C	109.5
C4—C3—C2	120.57 (17)	H16A—C16—H16B	109.5
C4—C3—H3	119.7	H16A—C16—H16C	109.5
C3—C4—C5	120.76 (17)	H16B—C16—H16C	109.5
C3—C4—H4	119.6	N2—C17—C18	113.83 (15)
C5—C4—H4	119.6	N2—C17—H17A	108.8
C4—C5—C8	120.76 (17)	N2—C17—H17B	108.8
C6—C5—C4	118.59 (16)	C18—C17—H17A	108.8
C6—C5—C8	120.65 (17)	C18—C17—H17B	108.8
C5—C6—H6	119.6	H17A—C17—H17B	107.7
C7—C6—C5	120.79 (17)	C17—C18—H18A	109.5
C7—C6—H6	119.6	C17—C18—H18B	109.5
C2—C7—H7	119.7	C17—C18—H18C	109.5
C6—C7—C2	120.57 (16)	H18A—C18—H18B	109.5
C6—C7—H7	119.7	H18A—C18—H18C	109.5
C5—C8—H8A	109.5	H18B—C18—H18C	109.5
			
O4—Co1—O2—C1	163.36 (13)	C15—N2—C17—C18	100.98 (19)
O4^i^—Co1—O2—C1	−16.64 (13)	O1—C1—C2—C7	176.64 (15)
N1—Co1—O2—C1	76.73 (13)	O2—C1—C2—C7	−3.4 (2)
N1^i^—Co1—O2—C1	−103.27 (13)	O1—C1—C2—C3	−3.4 (2)
O2—Co1—N1—C9	−147.01 (13)	O2—C1—C2—C3	176.48 (15)
O2^i^—Co1—N1—C9	32.99 (13)	C1—C2—C3—C4	177.72 (15)
O4—Co1—N1—C9	124.83 (13)	C7—C2—C3—C4	−2.4 (3)
O4^i^—Co1—N1—C9	−55.17 (13)	C5—C4—C3—C2	0.6 (3)
O2—Co1—N1—C13	32.98 (13)	C3—C4—C5—C6	1.3 (3)
O2^i^—Co1—N1—C13	−147.02 (13)	C3—C4—C5—C8	−178.90 (18)
O4—Co1—N1—C13	−55.18 (13)	C7—C6—C5—C4	−1.6 (3)
O4^i^—Co1—N1—C13	124.82 (13)	C7—C6—C5—C8	178.64 (18)
Co1—O2—C1—O1	31.7 (2)	C6—C7—C2—C1	−177.98 (16)
Co1—O2—C1—C2	−148.20 (11)	C6—C7—C2—C3	2.1 (2)
Co1—N1—C9—C10	178.06 (12)	C2—C7—C6—C5	−0.1 (3)
C13—N1—C9—C10	−1.9 (2)	N1—C9—C10—C11	0.3 (3)
Co1—N1—C13—C12	−177.42 (13)	C12—C11—C10—C9	0.8 (3)
C9—N1—C13—C12	2.6 (2)	C13—C12—C11—C10	−0.2 (2)
C15—N2—C14—O3	−175.13 (16)	C14—C12—C11—C10	171.09 (16)
C15—N2—C14—C12	5.7 (2)	C11—C12—C13—N1	−1.5 (3)
C17—N2—C14—O3	4.4 (2)	C14—C12—C13—N1	−173.31 (15)
C17—N2—C14—C12	−174.73 (15)	C11—C12—C14—O3	−118.02 (19)
C14—N2—C15—C16	116.2 (2)	C11—C12—C14—N2	61.2 (2)
C17—N2—C15—C16	−63.3 (2)	C13—C12—C14—O3	53.4 (2)
C14—N2—C17—C18	−78.6 (2)	C13—C12—C14—N2	−127.43 (17)

Symmetry codes: (i) −*x*, −*y*, −*z*.

### Hydrogen-bond geometry (Å, °)

**Table:**

Cg1 is the centroid of the C2–C7 ring.

**Table:**

*D*—H···*A*	*D*—H	H···*A*	*D*···*A*	*D*—H···*A*
O4—H41···O1^ii^	1.00 (2)	1.69 (2)	2.6443 (18)	160 (3)
O4—H42···O3^iii^	0.89 (2)	1.88 (2)	2.7557 (18)	170 (2)
C6—H6···O1^iv^	0.93	2.40	3.249 (3)	152
C11—H11···O1^v^	0.93	2.42	3.339 (2)	168
C17—H17A···Cg1^vi^	0.97	2.95	3.594 (2)	125

Symmetry codes: (ii) −*x*, −*y*, −*z*; (iii) −*x*+1, −*y*, −*z*; (iv) ; (v) ; (vi) *x*−1, *y*+1, *z*.

## References

[bb1] Bigoli, F., Braibanti, A., Pellinghelli, M. A. & Tiripicchio, A. (1972). *Acta Cryst.* B**28**, 962–966.

[bb2] Bruker (2005). *SADABS* Bruker AXS Inc., Madison, Wisconsin, USA.

[bb3] Bruker (2007). *APEX2* and *SAINT* Bruker AXS Inc., Madison, Wisconsin, USA.

[bb4] Farrugia, L. J. (1999). *J. Appl. Cryst.* **32**, 837–838.

[bb5] Hökelek, T., Dal, H., Tercan, B., Özbek, F. E. & Necefoğlu, H. (2009*a*). *Acta Cryst.* E**65**, m466–m467. 10.1107/S1600536809011209 PMC2968958 21582397

[bb6] Hökelek, T., Dal, H., Tercan, B., Özbek, F. E. & Necefoğlu, H. (2009*b*). *Acta Cryst.* E**65**, m513–m514. 10.1107/S160053680901318X PMC2977573 21583759

[bb7] Hökelek, T., Dal, H., Tercan, B., Özbek, F. E. & Necefoğlu, H. (2009*c*). *Acta Cryst.* E**65**, m607–m608. 10.1107/S1600536809015645 PMC2977639 21583825

[bb8] Hökelek, T., Gündüz, H. & Necefoğlu, H. (1996). *Acta Cryst.* C**52**, 2470–2473.

[bb9] Hökelek, T. & Necefoğlu, H. (1998). *Acta Cryst.* C**54**, 1242–1244.

[bb10] Krishnamachari, K. A. V. R. (1974). *Am. J. Clin. Nutr.* **27**, 108–111. 10.1093/ajcn/27.2.108 4812927

[bb11] Macrae, C. F., Edgington, P. R., McCabe, P., Pidcock, E., Shields, G. P., Taylor, R., Towler, M. & van de Streek, J. (2006). *J. Appl. Cryst.* **39**, 453–457.

[bb12] Necefoğlu, H., Çimen, E., Tercan, B., Ermiş, E. & Hökelek, T. (2010). *Acta Cryst.* E**66**, m361–m362. 10.1107/S1600536810007385 PMC2984063 21580476

[bb13] Sheldrick, G. M. (2008). *Acta Cryst.* A**64**, 112–122. 10.1107/S0108767307043930 18156677

[bb14] Spek, A. L. (2009). *Acta Cryst.* D**65**, 148–155. 10.1107/S090744490804362X PMC2631630 19171970

